# Supplementary material for: A highly energy-efficient multi-core neuromorphic architecture for training deep spiking neural networks
Source: Nat Commun. 2026 Mar 25;17:4403. doi: 10.1038/s41467-026-70586-x (PMC13181023; doi:10.1038/s41467-026-70586-x)
Supplement: Supplementary file 1 — Supplementary Information [file 41467_2026_70586_MOESM1_ESM.pdf]

Supplementary Information for: A Highly Energy-Efficient Multi-Core Neuromorphic Architecture for Training Deep Spiking Neural Networks

Mingjing Li[†,1], Huihui Zhou[†,*,1,2,3], Xiaofeng Xu[†,1], Zhiwei Zhong[†,1], Puli Quan[1], Xueke Zhu[1], Yanyu Lin[1], Wenjie Lin[1], Xiaosha Li[1], Dong Wang[1], Junchao Zhang[1], Yunhao Ma[1,4], Xiaole Cui[1,5], Wei Wang[1], Qingyan Meng[1], Zhengyu Ma[1], Guoqi Li[*,1,6], Xiaoxin Cui[*,1,7], Yonghong Tian[*,1,2,3]

[†] These authors contributed equally

[*] Corresponding authors, emails: zhouhui_h@qq.com, guoqi.li@ia.ac.cn, cuixx@pku.edu.cn, yhtian@pku.edu.cn

1. Pengcheng Laboratory, Shenzhen, China
2. School of AI for Science, Peking University Shenzhen Graduate School, Shenzhen, China
3. Beijing Key Laboratory of Brain-inspired Spiking Large Models, School of Computer Science, Peking University, Beijing, China
4. Southern University of Science and Technology, Shenzhen, China
5. School of Electronics and Computer Engineering, Peking University Shenzhen Graduate School, Shenzhen, China
6. Institute of Automation, Chinese Academy of Sciences, Beijing, China
7. School of Integrated Circuits, Peking University, Beijing, China

1. Supplementary Content 1. Comparison of Intermediate Representations in SNN Representation and Mapping

Open-source neuromorphic software frameworks such as Norse [1], SpikingJelly [2], snnTorch [3], Sinabs [4], and Spyx [5] are built upon PyTorch or JAX and share the intermediate representations (IRs) with these mainstream deep learning frameworks. PyTorch and JAX IRs are primarily targeted at artificial neural networks (ANNs) [6]. These open-source IRs using basic operations, such as multiplications, additions, and reshaping, enable flexibility in mapping and various compiler-level optimizations. Meanwhile, these IRs lack the explicit representation of the temporal dimension and spiking behavior of spiking neurons in SNN [6], rendering the SNN representation based on these IRs complex. For example, in the PyTorch IR representation of a simple two-time-step Leaky Integrate-and-Fire (LIF) neuron followed by convolution (Suppl. Figure 8a), crucial components like time-step indexing and spiking behaviors of the neuron are difficult to be extracted and mapped to the neuromorphic architectures with dedicated hardware to support computation through time steps and spiking mechanisms. Our custom IR introduces a dedicated Soma operation (Suppl. Figure 8b) that concisely encapsulates all key aspects of the LIF neuron, resulting in its straightforward mapping to the Soma module (Figure 2c) mainly through setting these parameters shown in the Soma operation (Suppl. Figure 8b) in corresponding registers in our neuromorphic architecture. We also compare the size between PyTorch, JAX, and our custom IRs in representing deep SNNs (Supplementary Table 8a), showing that representations based on our IR are clearly smaller than those based on PyTorch IR and JAX IR. We further compare PyTorch and JAX IRs in representing ANNs and SNNs, showing that PyTorch and JAX IRs are more efficient for the ANN representation (Supplementary Table 8b).

On the other hand, neuromorphic software tools such as PyNN [7], NEST [8], Nengo [9], and NEURON [10] are primarily designed for simulating biological neural networks. Tools like Lava [11], Corelet [12], and Rockpool [13] support the SNN representation for specific neuromorphic hardware platforms such as Loihi [14,15], TrueNorth [16], and Xylo [17].

Recently, Pedersen et al. [6] proposed a general-purpose Neuomorphic IR (NIR). NIR shares a similar level of operand abstraction as our custom IR, which also encapsulates components of the neuron model into one single operation. The optimization of operand abstraction of IRs might improve SNN representation and mapping. For example, IRs with more primitive operations and explicit representation of the temporal dimension and spiking behavior might enable various compiler-level optimizations and straightforward mapping to neuromorphic architectures.

2. Supplementary Figure 1

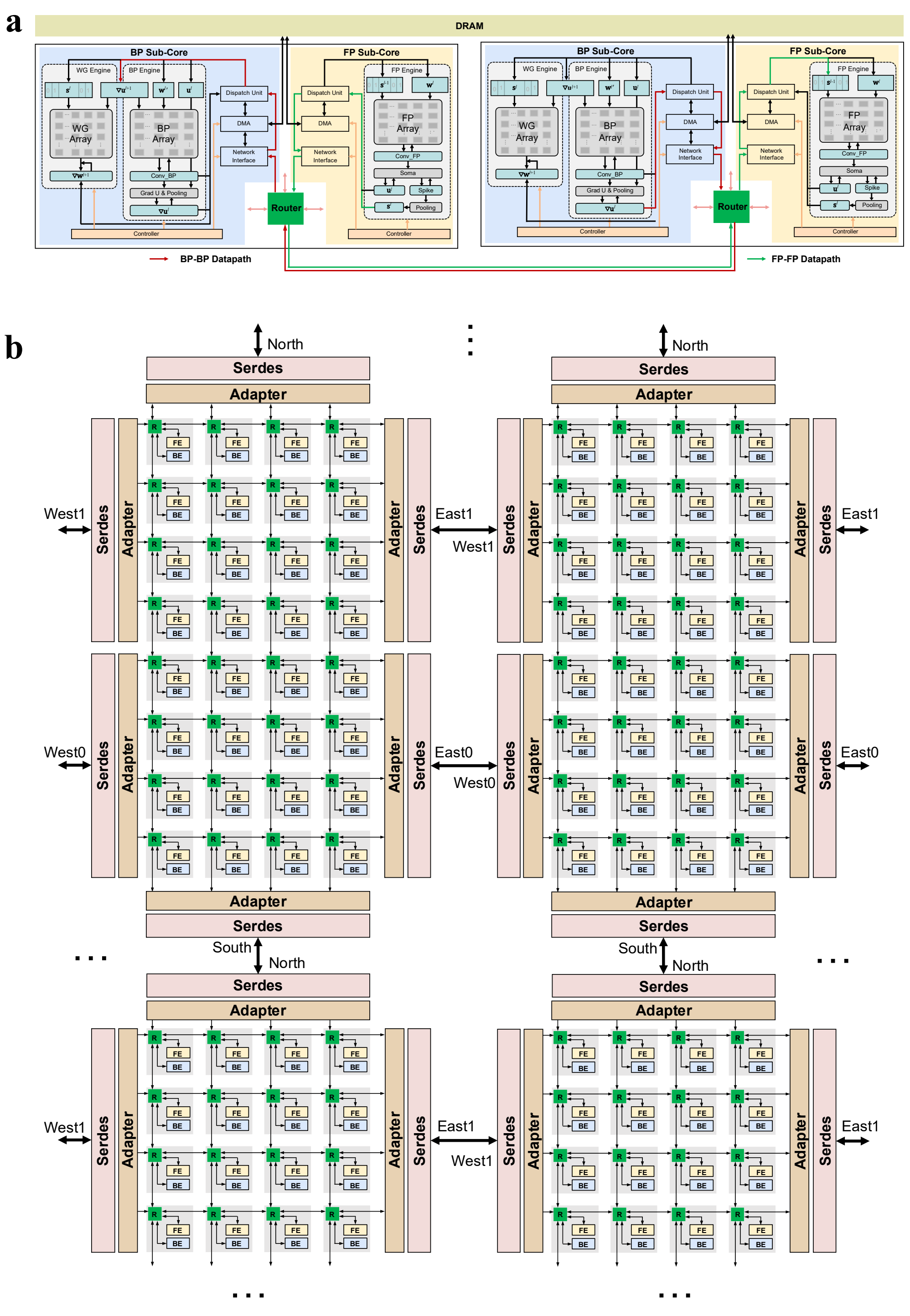

Supplementary Figure 1. Data transmission of the multi-core neuromorphic architecture. (a) Intra- and inter-core data transmission pathways in the multi-core architecture. Green lines represent the data transmission pathway in forward computation, while red lines represent the pathway in backpropagation. Intra-core FP-BP transmission is through the pathway FP sub-core SRAM → DU → NI → Router→ NI → DU → target BP sub-core SRAM within the same core. b) Inter-chip communication design. The 2D mesh NOC network in our 32-core architecture can be extended in four directions through the six custom Serdes to support high inter-chip scalability. Our architecture supports extension in the east-west direction via two Serdes links with 8 ports, and in the north-south direction via one SerDes link with 4 ports. Thus, adjacent chips can directly interface across chip boundaries, thereby supporting chip tiling and high chip-level scalability.

3. Supplementary Figure 2

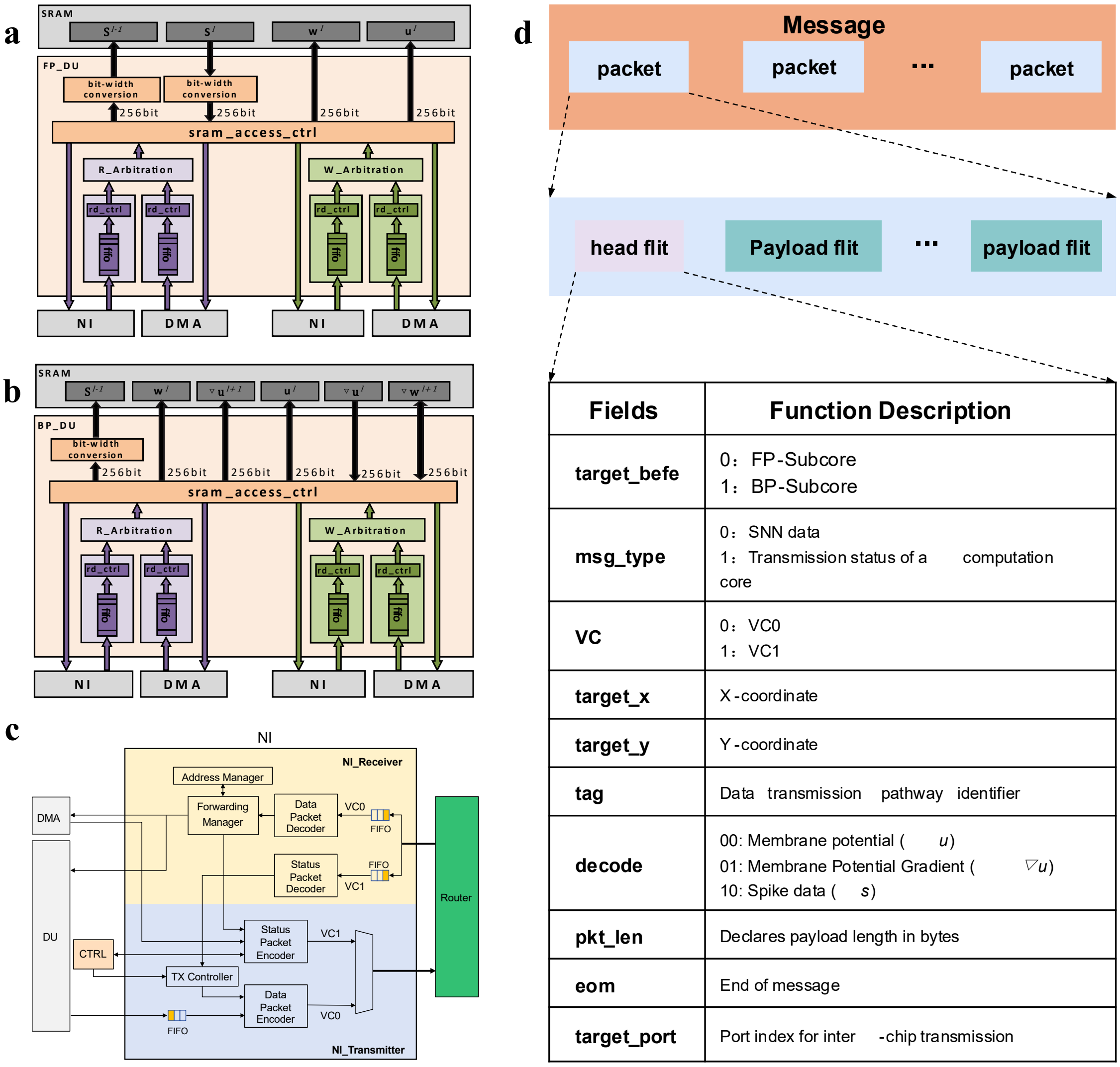

Fields	Function Description
target_befe	0：FP-Subcore<br>1：BP-Subcore
msg_type	0：SNN data<br>1：Transmission status of a computation core
VC	0：VC0<br>1：VC1
target_x	X-coordinate
target_y	Y-coordinate
tag	Data transmission pathway identifier
decode	00: Membrane potential (u)<br>01: Membrane Potential Gradient (∇u)<br>10: Spike data (s)
pkt_len	Declares payload length in bytes
eom	End of message
target_port	Port index for inter -chip transmission

Supplementary Figure 2. NoC modules and transmission data Packet design. (a) The FP Dispatch Unit (DU) module serves as a data distribution hub that manages the input and output of SRAMs used for the FP Engine. (b) The BP Dispatch Unit (DU) module serves as a data distribution hub that manages the input and output of SRAMs used for the BP Engine. (c) Network Interface (NI) module. The NI transmitter fetches data payloads from SRAMs and encapsulates them into packets according to the head flit structure, dynamically regulating transmission timing through flow control status. The NI receiver performs packet decapsulation, resolves destination-aware write operations, and manages address mapping information for SRAM/DDR interfaces. (d) The structure of messages, packets, and head flits, with a functional description of each field in the head flit.

4. Supplementary Figure 3

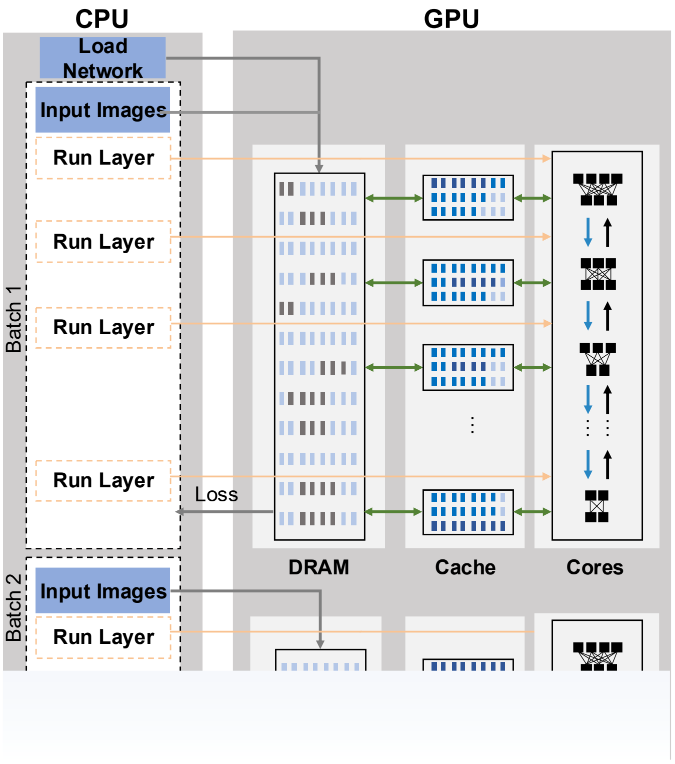

Supplementary Figure 3. The training procedure in a GPU system.

5. Supplementary Figure 4

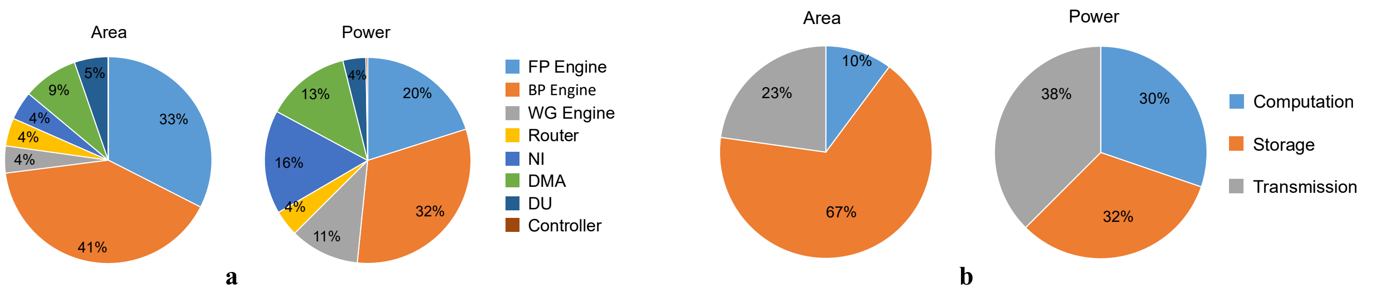

Supplementary Figure 4. Area and power estimation based on RTL simulation. (a) Estimation of area and power consumption of modules in a computing core. (b) Area and power breakdown of computation, storage, and transmission.

6. Supplementary Figure 5

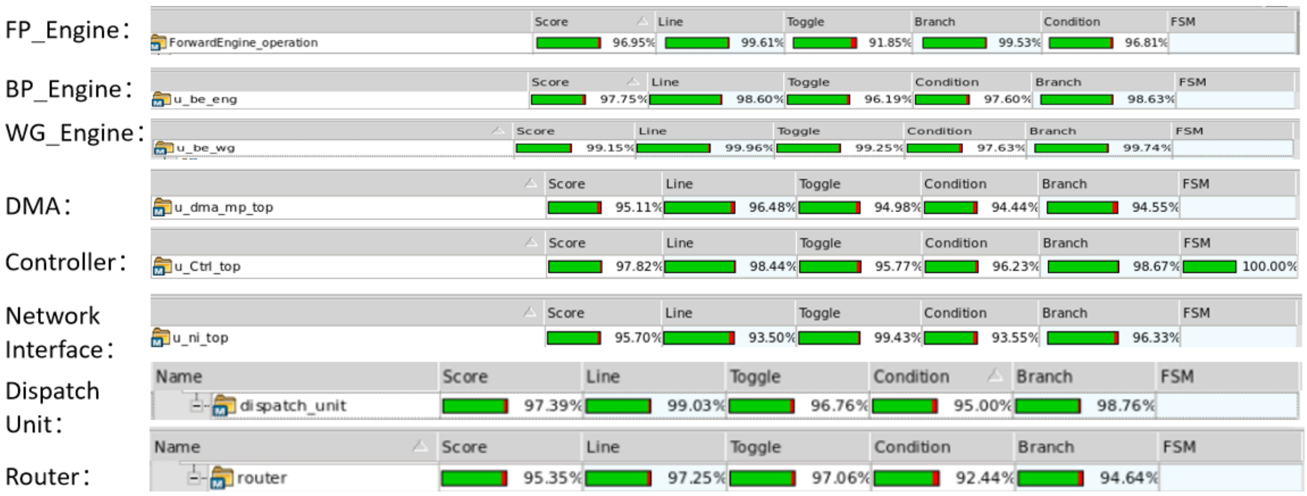

Supplementary Figure 5. The code coverage of modules. Code coverage scores of FP engine, BP engine, and WG engine are 96.95%, 97.75%, and 99.15%, respectively. Code coverage scores of NOC are 95.11% (DMA), 97.82% (Controller), 95.70% (NI), 97.39% (DU), and 95.35% (Router), respectively.

7. Supplementary Figure 6

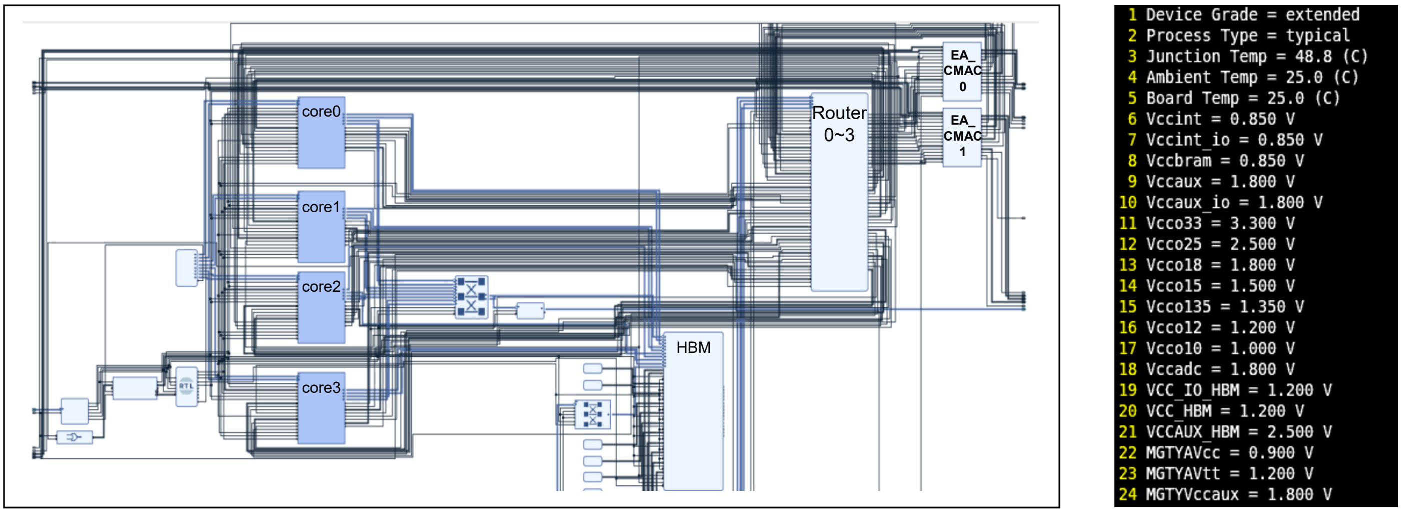

Supplementary Figure 6. FPGA block design and operating conditions. (a) Partial FPGA Block Design of Multi-Core Architecture. (b) FPGA operating condition report.

8. Supplementary Figure 7

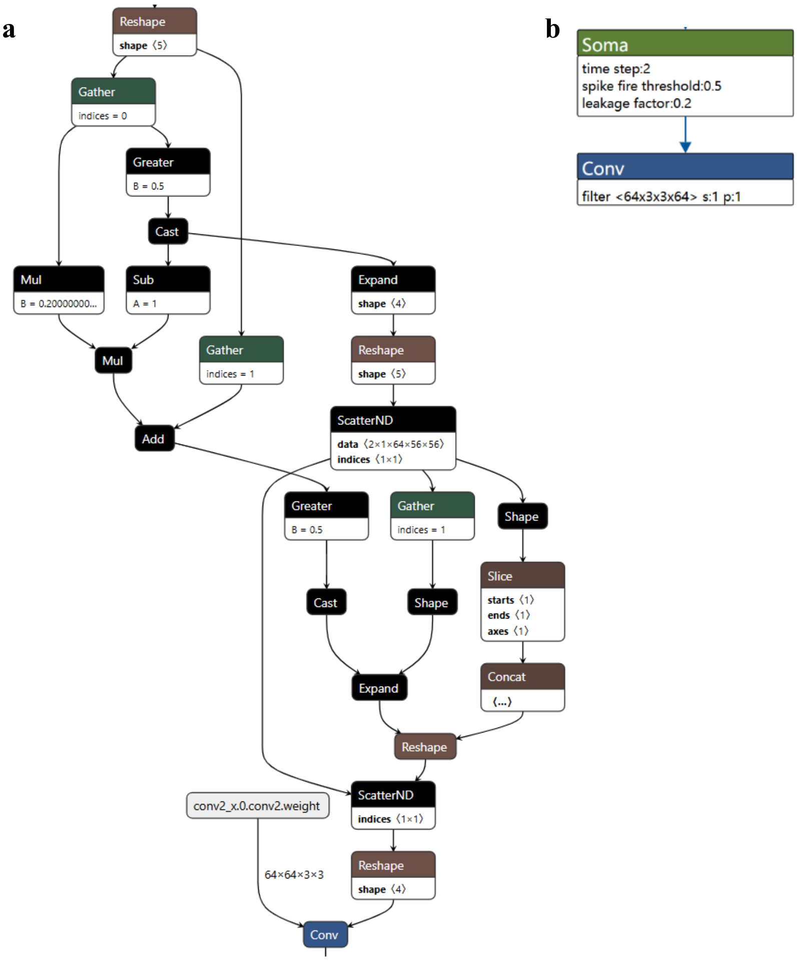

Supplementary Figure 7. Intermediate Representation (IR) of SNN. (a) PyTorch IR for representing a two-time-step LIF model; (b) Our IR for representing a two-time-step LIF model.

9. Supplementary Figure 8

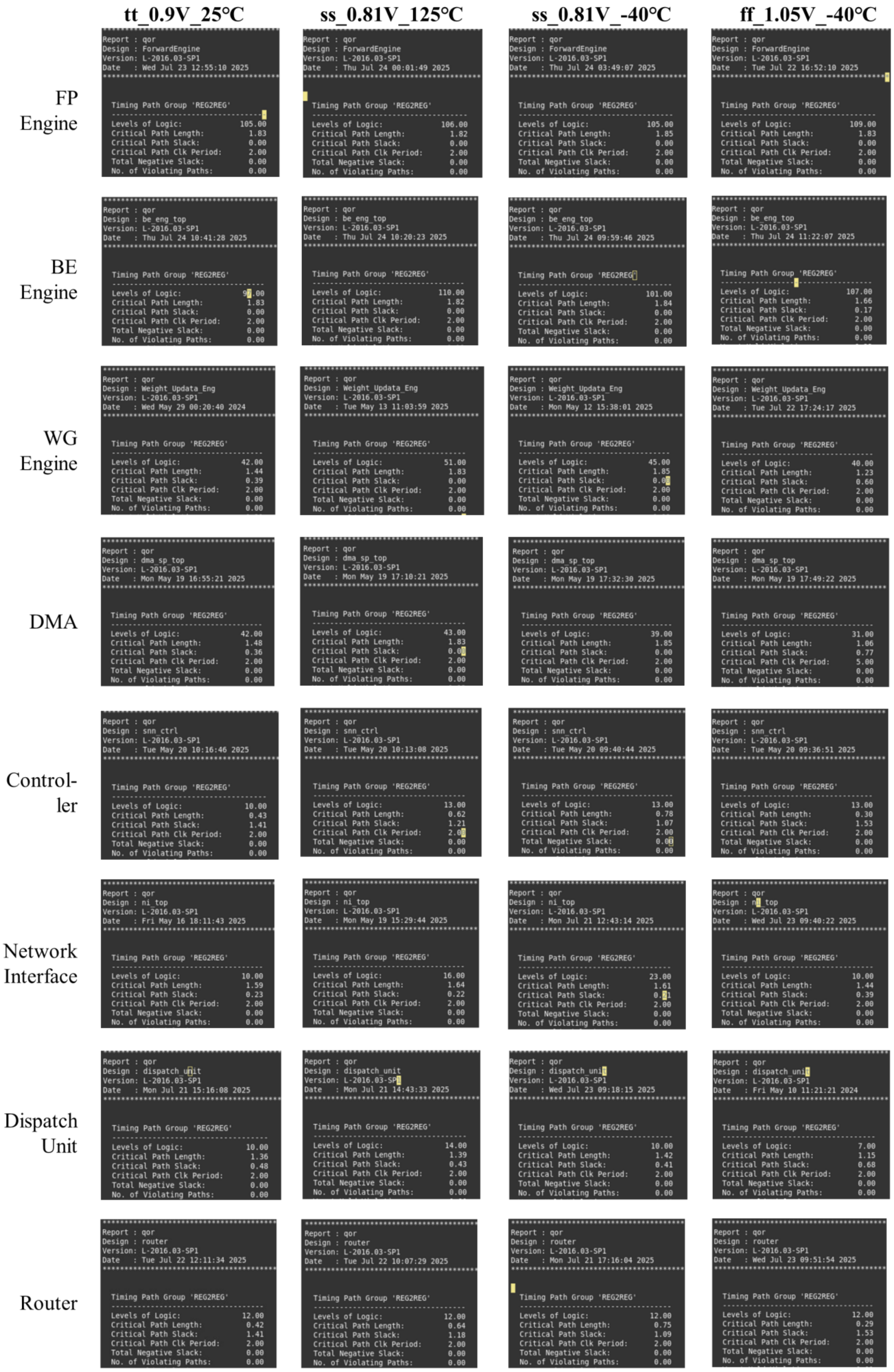

Supplementary Figure 8. Timing closure analysis of modules in our architecture.

10. Supplementary Figure 9

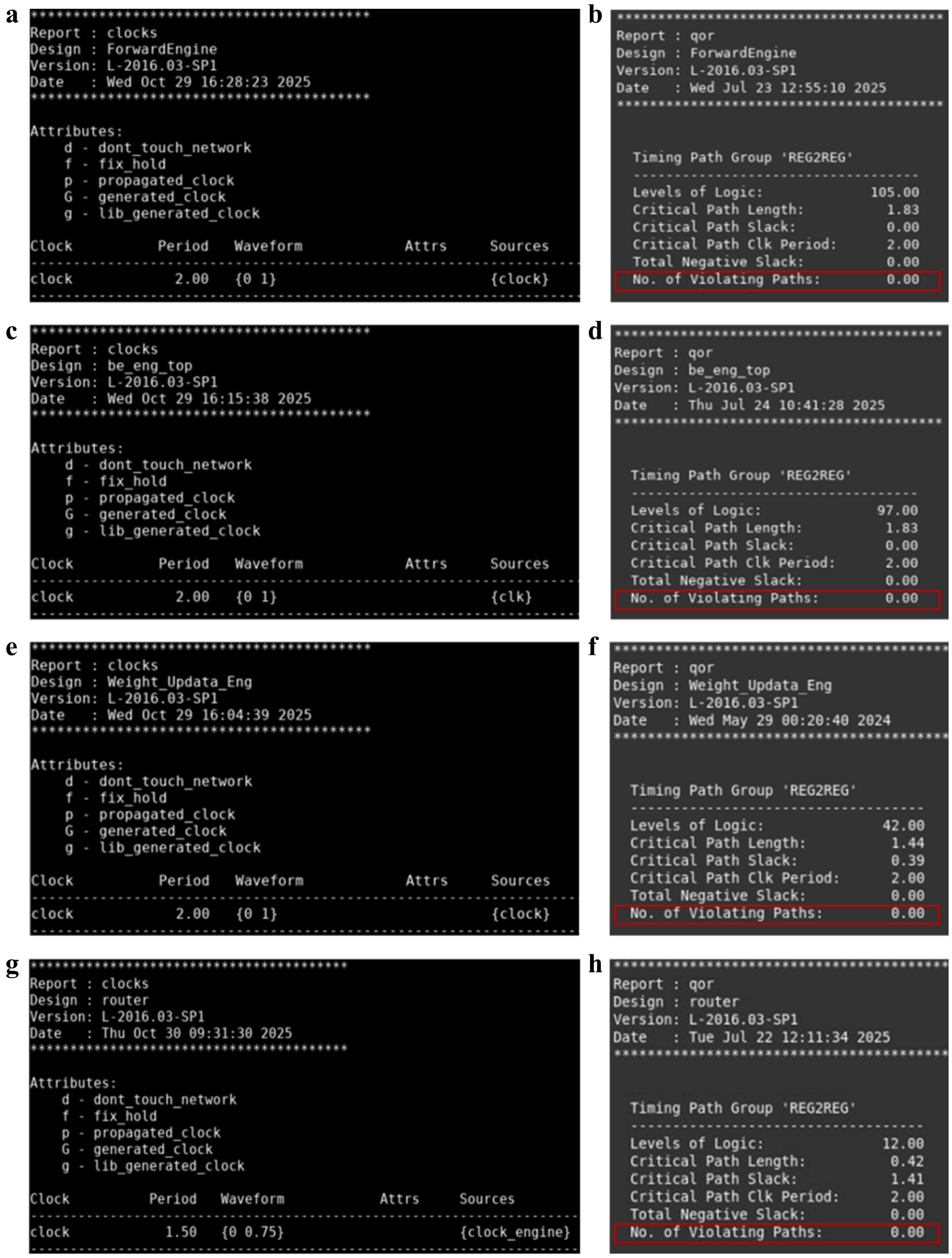

Supplementary Figure 9. **The clock frequency setting and time closure report in the performance evaluation.** (a) The clock frequency setting of the FP Engine. (b) Time closure report of the FP Engine. (c) The clock frequency setting of the BP Engine. (d) Time closure report of the BP Engine. (e) The clock frequency setting of the WG Engine. (f) Time closure report of the WG Engine. (g) The clock frequency setting of the Router. (h) Time closure report of the Router.

11. Supplementary Figure 10

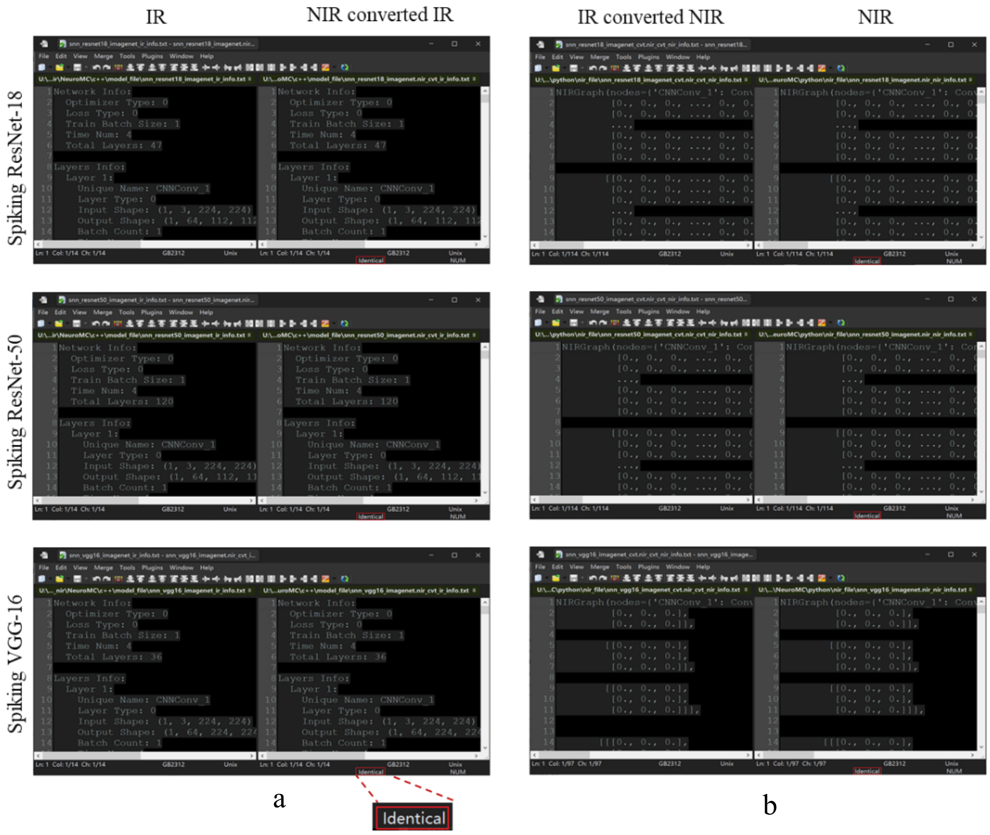

Supplementary Figure 10. **Bidirectional conversion between our proprietary IR format and the NIR format.** (a) The IRs of Spiking ResNet-18, -50, and Spiking VGG-16 models converted from the NIR framework (version 1.0.6) are identical to those IRs directly exported from our software toolchain. (b) The NIRs of those models converted from our IRs are identical to the NIRs directly exported from the NIR framework. “Identical” indicates that the data in the two files is an exact match, as determined by Notepad++’s Compare plugin.

12. Supplementary Algorithm 1

Supplementary Algorithm 1 Design of Parallelized SNN Training Operations in the Multi-core Neuromorphic Architecture

1: N, L; % N: batch size; L: SNN layer number — $l \in \{0, \cdots, L-1\}$; % Layer index
2: T; % Total number of time steps — $t \in \{0, \cdots, T-1\}$; % Index of a time step
3: Core_Num = 32; % Total number of computing cores in the architecture — $k \in \{0, \cdots,$ Core_Num$-1\}$; % Index of a computing core
4: Engine_Num = 3; % Total number of computing engines in a core — $\mathbf{e}_i \in \{0, \cdots,$ Engine_Num$-1\}$; % Index of a computing engine
5: **n**= zero(1, Core_Num); % Indices of samples processed in FP engines — **n_bp** = zero(1, Core_Num); % Indices of samples processed in BP engines
6: **n_wg** = zero(1, Core_Num); % Indices of samples processed in WG engines — α; % Leakage factor

7: parallel for $k = 0$: Core_Num-1 do % parallel computation across computing cores
8: parallel for $\mathbf{e}_i = 0$: Engine_Num-1 do % parallel computation across computing engines
9: if $\mathbf{e}_i == 0$ % computation in the FP engine
10: Read $\mathbf{w}_k^l$ from SRAM; % $\mathbf{w}_k^l$: weights of layer l in core k
11: for $t = 0 : T-1$ do % forward computation along time steps
12: While ∼exist($\mathbf{s}^{l-1}_{\mathbf{n}[k],t}$) wait; % wait until spike input from layer l-1 arrives
13: Read $\mathbf{s}^{l-1}_{\mathbf{n}[k],t}$ from SRAM % $\mathbf{s}^{l-1}_{\mathbf{n}[k],t}$: spikes input from layer l-1 at time step t of sample $\mathbf{n}[k]$
14: $\mathbf{Conv\_FP}^l_{\mathbf{n}[k],t}[i] = \sum \mathbf{s}^{l-1}_{\mathbf{n}[k],t}[i]\mathbf{w}^l_k[j,i]$; % convolution computation between spike input from layer l-1 and weights of layer l
15: if $t == 0$ $\mathbf{u}^l_{\mathbf{n}[k],t-1}[i] = 0$; endif % initialize membrane potential when $t = 0$
16: $\mathbf{u}^l_{\mathbf{n}[k],t}[i] = \alpha \mathbf{u}^l_{\mathbf{n}[k],t-1}[i](1 - \mathbf{s}^l_{\mathbf{n}[k],t-1}[i]) + \mathbf{Conv\_FP}^l_{\mathbf{n}[k],t}[i]$; % update the membrane potential at time step t
17: $\mathbf{s}^l_{\mathbf{n}[k],t}[i] = \mathrm{fire}(\mathbf{u}^l_{\mathbf{n}[k],t}[i])$; %generate spike output of layer l at time step t
18: Send $\mathbf{s}^l_{\mathbf{n}[k],t}$ to current FP engine; % data dispatching for upcoming computation of **Conv_FP** of layer l in next time step
19: Send $\mathbf{s}^l_{\mathbf{n}[k],t}$ to another FP engine; % data dispatching for upcoming computation of **Conv_FP** of layer l+1 in another core
20: Send $\mathbf{s}^l_{\mathbf{n}[k],t}$,$\mathbf{u}^l_{\mathbf{n}[k],t}$ to BP and WG engines in the same core or DRAM; % data dispatching for upcoming BP and WG computation
21: endfor % end of FP computation across time steps
22: if $l == L-1$ and $t == T-1$ calculate error $\mathbf{E}[\mathbf{n}[k]]$; endif % computing calculate error when FP computation in the last layer is finished
23: if $\mathbf{n}[k] < N-1$ $\mathbf{n}[k] = \mathbf{n}[k] + 1$; goto Line 11; endif % update sample index and go to Line 11 to process the next sample
24: endif % end of computation in the FP engine

25: if $\mathbf{e}_i == 1$ % computation in the BP engine
26: Read $\mathbf{w}_k^{l+1}$ from SRAM % read weights of layer l+1 to BP Array in core k
27: for $t = T-1 : 0$ do % backward computation along time steps
28: While ∼exist($\nabla \mathbf{u}^{l+1}_{\mathbf{n\_bp}[k],t}$) wait; % wait until membrane potential gradients of layer l+1 arrive
29: Read $\nabla \mathbf{u}^{l+1}_{\mathbf{n\_bp}[k],t}$ from SRAM; % $\nabla \mathbf{u}^{l+1}_{\mathbf{n\_bp}[k],t}$: membrane potential gradients of layer l+1 at time step t of sample $\mathbf{n\_bp}[k]$
30: Read $\mathbf{s}^l_{\mathbf{n\_bp}[k],t}$, $\mathbf{u}^l_{\mathbf{n\_bp}[k],t}$ from SRAM;
31: $\mathbf{Conv\_BP}^l_{\mathbf{n\_bp}[k],t}[i] = \sum \nabla \mathbf{u}^{l+1}_{\mathbf{n\_bp}[k],t}[j]\mathbf{w}^{l+1}_k[i,j]$; % convolution computation between membrane potential gradients and weights from layer l+1
32: calculate $\mathrm{fire}'(\mathbf{u}^l_{\mathbf{n\_bp}[k],t}[i])$; % calculate the surrogate gradient of the spike function
33: if $\mathrm{fire}'(\mathbf{u}^l_{\mathbf{n\_bp}[k],t}[i])\ != 0$ % $\nabla \mathbf{s}^l_{\mathbf{n\_bp}[k],t}[i]$: spike gradients of layer l at time step t of the sample $\mathbf{n\_bp}[k]$
34: $\nabla \mathbf{s}^l_{\mathbf{n\_bp}[k],t}[i] = -\alpha \nabla \mathbf{u}^l_{\mathbf{n\_bp}[k],t+1}[i]\mathbf{u}^l_{\mathbf{n\_bp}[k],t}[i] + \mathbf{Conv\_BP}^l_{\mathbf{n\_bp}[k],t}[i]$; % compute the spike gradients of layer l at time step t
35: $\nabla \mathbf{u}^l_{\mathbf{n\_bp}[k],t}[i] = \alpha \nabla \mathbf{u}^l_{\mathbf{n\_bp}[k],t+1}[i](1 - \mathbf{s}^l_{\mathbf{n\_bp}[k],t}[i]) + \nabla \mathbf{s}^l_{\mathbf{n\_bp}[k],t}[i]\mathrm{fire}'(\mathbf{u}^l_{\mathbf{n\_bp}[k],t}[i])$;
36: else $\nabla \mathbf{u}^l_{\mathbf{n\_bp}[k],t}[i] = \alpha \nabla \mathbf{u}^l_{\mathbf{n\_bp}[k],t+1}[i](1 - \mathbf{s}^l_{\mathbf{n\_bp}[k],t}[i])$; % compute membrane potential gradients of layer l at time step t
37: endif
38: Send $\nabla \mathbf{u}^l_{\mathbf{n\_bp}[k],t}$ to current BP engine; % data dispatching for upcoming computation of the gradients at time step t-1
39: Send $\nabla \mathbf{u}^l_{\mathbf{n\_bp}[k],t}$ to WG engine in another core; % data dispatch for upcoming computation of weight gradients of layer l
40: Send $\nabla \mathbf{u}^l_{\mathbf{n\_bp}[k],t}$ to BP engine in another core; % data dispatching for upcoming computation of **Conv_BP** of layer l-1
41: endfor % end of BP computation across time steps
42: if $\mathbf{n\_bp}[k] < N$ $\mathbf{n\_bp}[k] = \mathbf{n\_bp}[k] + 1$; goto Line 27; endif % update sample index and go to Line 27 to process the next sample
43: endif % end of computation in the BP engine

44: if $\mathbf{e}_i == 2$ % Computation in the WG engine
45: for $t = T-1 : 0$ do % backward computation along time steps
46: While ∼exist($\nabla \mathbf{u}^{l+1}_{\mathbf{n\_wg}[k],t}$) wait; % wait until membrane potential gradients of layer l+1 arrive
47: Read $\nabla \mathbf{u}^{l+1}_{\mathbf{n\_wg}[k],t}$ from SRAM;
48: Read $\mathbf{s}^l_{\mathbf{n\_wg}[k],t}$ from SRAM;
49: $\nabla \mathbf{w}^{l+1}[i,j] = \sum_t \nabla \mathbf{u}^{l+1}_{\mathbf{n\_wg}[k],t}[j]\mathbf{s}^l_{\mathbf{n\_wg}[k],t}[i]$; % integrate weight gradients of layer l+1 across time steps
50: endfor % end of WG computation across time steps
51: $\nabla \mathbf{w}^{l+1} = \nabla \mathbf{w}^{l+1}/T$; % generate weight gradients of layer l+1
52: if $\mathbf{n\_wg}[k] < N$ $\mathbf{n\_wg}[k] = \mathbf{n\_wg}[k] + 1$; goto Line 45; % update sample index and go to Line 45 to process the next sample
53: else $\mathbf{w}^{l+1} = \mathbf{w}^{l+1} + \mathrm{d}\mathbf{w}^{l+1}$; % Update weights of layer l+1 after processing a batch of samples
54: endif
55: endif % end of computation in the WG engine
56: endfor % end of parallel for $\mathbf{e}_i = 0$: Engine_Num-1
57: endfor % end of parallel for $k = 0$: Core_Num-1

The algorithm describes the parallelized operations for one batch of training in the multi-core neuromorphic architecture. During SNN training, an SNN model is deployed in a distributed manner across all computing cores of our multi-core architecture, with one layer or a segment of a layer allocated to an engine within a computing core. Within the same core, without loss of generality, the FP engine computes the spike outputs and membrane potentials of layer l, and the BP engine calculates the membrane potential gradients of layer l, while the WG engine computes the weight gradients of layer l+1. Prior to training, all sample indices are set to 0 for all engines (Lines 5-6), indicating that all engine computations start from the first sample. Our architecture supports both inter-core parallelism (Line 7) and intra-core engine parallelism (Line 8) computations:

The FP engine computes spike outputs and membrane potentials of layer l neurons (Lines 14-17) along time steps (Line 11), after ensuring correct spike inputs from layer l-1 (Line 12). The computed results are dispatched (Lines 18-20) for subsequent FP computations of layer l at the next time step (Line 18), for FP computations of layer l+1 at the current time step (Line 19), and for future BP/WG computations (Line 20). After completing computations along all time steps, the FP engine processes the next sample (Line 23). The architecture supports FP computations of different samples, layers, and time steps simultaneously across computing cores (Line 7).

The BP engine calculates membrane potential gradients of layer l (Lines 31-36) in reverse time order (Line 27), after ensuring correct potential gradient inputs from layer l+1 (Line 28). The computed results are dispatched (Lines 38-40) for upcoming BP calculations of layer l at the preceding time step (Line 38), for WG computations of layer l (Line 39), and for BP computations of layer l-1 at the current time step (Line 40). Upon completing computations along all time steps, the BP engine proceeds with the next sample (Line 42). Within the same core, FP and BP engines compute spike outputs, membrane potentials (Lines 16-17) and membrane potential gradients (Lines 35-36) of the same model layer (l) across different samples and time steps simultaneously (Line 8). The architecture also supports BP computations of different samples, layers, and time steps simultaneously across computing cores (Line 7).

The WG engine computes weight gradients of layer l+1 in reverse time order (Line 45), after ensuring correct potential gradient inputs from layer l+1 (Line 46). Upon completing computations along all time steps, the WG engine processes the next sample (Line 52). Within the same core, the WG engine computes weight gradients of layer l+1 (Line 49) in parallel (Line 8) with the computations in the FP (Lines 16-17) and BP (Lines 35-36) engines. The architecture also supports WG computations of different samples, layers, and time steps simultaneously across computing cores (Line 7).

Together, our multi-core architecture supports parallel execution of FP, BP, and WG computations across different samples, model layers, and time steps simultaneously in different cores. Within the same core, FP and BP engines compute spike outputs, membrane potentials and membrane potential gradients of the same SNN layer (l) across different samples and time steps simultaneously, and WG engine computes weight gradients of layer l+1 in parallel with the computations in the BP and FP engines. Thus, the architecture supports highly parallel computing at both the core and engine levels during SNN training.

13. Supplementary Table 1

Instruction Set

Opcode	Operand					
FP_CONV	s_h_size	s_w_size	k_size	padding	stride	psum_acc
	c_size	m_size	c_offset	m_offset	Reserved	
	Compute convolution at FP ARRAY, fetch data from $\mathbf{s}^{l-1}$ and $\mathbf{w}^{l}$ from SRAM, refresh **Conv_FP**^{l} SRAM					
BP_CONV	du_h_size	du_w_size	k_size	padding	insert	psum_acc
	m_size	c_size	m_offset	c_offset	Reserved	
	Compute convolution at BP ARRAY, fetch data from $\nabla\mathbf{u}^{l+1}$ and $\mathbf{w}^{l+1'}$ from SRAM, refresh **Conv_BP**^{l} SRAM					
WG_CONV	s_h_size	s_w_size	du_h_size	du_w_size	Insert	dw_acc
	dw_c_size	dw_m_size	dw_c_offset	dw_m_offset	Reserved	
	Compute convolution at WG ARRAY, fetch data from $\mathbf{s}^{l}$ and $\nabla\mathbf{u}^{l+1}$ from SRAM, refresh $\nabla\mathbf{w}^{l+1}$ SRAM					
FP_SOMA	h_size	w_size	m_size	m_offset	pooling	t_acc
	Compute FP Soma, fetch data from **Conv_FP**^{l} 、$\mathbf{u}^{l}$、Spike from SRAM, refresh $\mathbf{s}^{l}$ SRAM					
BP_GRAD	h_size	w_size	c_size	c_offset	pooling	t_acc
	Compute BP Grad, fetch data from **Conv_BP**^{l} 、$\nabla\mathbf{u}^{l}$、$\mathbf{u}^{l}$、$\mathbf{s}^{l}$ 、**fire**' from SRAM, refresh $\nabla\mathbf{u}^{l}$ SRAM					
FP_BN	h_size	w_size	m_size	m_offset	acc	Reserved
	Compute BN at FP Engine, refresh **Conv_FP**^{l} SRAM and BN private buffer					
BP_BN	h_size	w_size	m_size	m_offset	acc	Reserved
	Compute BN at BP Engine, refresh $\nabla\mathbf{u}^{l+1}$ SRAM and BN private buffer					
FP_VECTOR	op_type	data_type	h_size	w_size	m_size	m_offset
	Compute VECTOR add/multiply/compare at FP Soma, refresh SRAM according to data_type, i.e. **Conv_FP**^{l} 、$\mathbf{s}^{l}$ SRAM					
BP_VECTOR	op_type	data_type	h_size	w_size	m_size	m_offset
	Compute VECTOR add/multiply/compare at BP Grad, refresh SRAM according to data_type, i.e. **Conv_BP**^{l} 、**fire**' SRAM					
NOC_DATA	flow_type	data_type	tag_id	Reserved		
	Send a message to NOC, fetch data from SRAM					
NOC_CTRL	flow_type	tag_id	msg_box			
	Send a control message to NOC, trig an event at destination					
DMA_WR	data_type	src_addr	dst_addr	length	Reserved	
	Move data from sram to dram					
DMA_RD	data_type	src_addr	dst_addr	length	Reserved	
	Move data from dram to sram					
BARRIER	sub_type	Reserved				
	BARRIER for instruction group					

14. Supplementary Table 2

Parameters of SNNs

Parameters	Description
N	batch size
T	time steps of SNN
H/W	input feature map height/width
C	input feature map channel
R/S	kernel height/width
M	output feature map channel
E/F	output feature map height/width

15. Supplementary Table 3

SRAMs of a Computing Core

FP-Subcore		BP-Subcore	
$\mathbf{s}^{l\text{-}1}$	32KB	$\nabla\mathbf{u}^{l+1}$	128KB
$\mathbf{w}^{l}$	576KB	$\mathbf{w}^{l+1\prime}$	576KB
**Conv_FP**^{l}	128KB	**Conv_BP**^{l}	128KB
$\mathbf{u}^{l}$	128KB	$\nabla\mathbf{u}^{l}$	128KB
$\mathbf{s}^{l}$	32KB	$\mathbf{u}^{l}$	128KB
Spike	32KB	$\mathbf{s}^{l}$	8KB
Others	16KB	$\nabla\mathbf{w}^{l+1}$	9 KB
		Others	24KB
Total	2073KB		

16. Supplementary Table 4

Comparisons of DRAM Occupancy during SNN Training

Model	Batch size	Architecture	Total Occupancy (GB)	Intermediate data (GB)	Optimizer state (GB)	Weight (GB)	Weight gradient(GB)
S-ResNet18	16	A100	2.01	1.45	0.42	0.07	0.07
		Ours	1.17 (-41.57%)	0.54 (-62.43%)	0.42	0.14	0.07
	32	A100	3.46	2.90	0.42	0.07	0.07
		Ours	1.72 (-50.31%)	1.09 (-62.40%)	0.42	0.14	0.07
	64	A100	6.35	5.79	0.42	0.07	0.07
		Ours	2.81 (-55.85%)	2.18 (-62.39%)	0.42	0.14	0.07
	128	A100	12.15	11.59	0.42	0.07	0.07
		Ours	4.99 (-58.96%)	4.36 (-62.39%)	0.42	0.14	0.07
S-ResNet50	16	A100	8.79	6.61	1.63	0.27	0.27
		Ours	4.87 (-44.62%)	2.42 (-63.39%)	1.63	0.54	0.27
	32	A100	15.4	13.22	1.63	0.27	0.27
		Ours	7.28 (-52.69%)	4.78 (-63.82%)	1.63	0.54	0.27
	64	A100	28.62	26.44	1.63	0.27	0.27
		Ours	12.12 (-57.65%)	9.68 (-63.41%)	1.63	0.54	0.27
	128	A100	55.06	52.89	1.63	0.27	0.27
		Ours	21.79 (-60.42%)	19.34 (-63.43%)	1.63	0.54	0.27
S-VGG16	16	A100	10.34	8.28	1.55	0.26	0.26
		Ours	4.15 (-59.81%)	1.83 (-77.89%)	1.55	0.52	0.26
	32	A100	18.62	16.56	1.55	0.26	0.26
		Ours	5.99 (-67.82%)	3.68 (-77.80%)	1.55	0.52	0.26
	64	A100	35.17	33.12	1.55	0.26	0.26
		Ours	9.66 (-72.53%)	7.34 (-77.84%)	1.55	0.52	0.26
	128	A100	68.28	66.25	1.55	0.26	0.26
		Ours	17.01 (-75.09%)	14.69 (-77.83%)	1.55	0.52	0.26

17. Supplementary Table 5

Sparsity of SNN Models

Models	Spike sparsity	Spike16 sparsity	*fire'* sparsity	∇u sparsity
S-ResNet18	90%	31%	68%	7%
S-ResNet50	80%	12%	54%	0%
S-VGG9	88%	25%	56%	62%

*The spike16 sparsity means portion of all 16 channles of spikes are zero in FP or WG computation.

18. Supplementary Table 6

Effects of Sparse Computation Optimization

Models	Conditions	FP (mW)	BP (mW)	WG (mW)	Total (mW)
S-ResNet18	no optimization	64.39	187.66	39.20	291.24
	with optimization	37.01	60.44	15.63	113.15
	Power decrease (%)	42.53%	67.74%	60.16%	61.09%
S-ResNet50	no optimization	64.38	187.66	39.20	291.24
	with optimization	46.95	86.63	21.13	154.71
	Power decrease (%)	27.01%	53.92%	46.24%	46.81%
S-VGG9	no optimization	64.38	187.66	39.20	291.24
	with optimization	40.45	82.89	17.05	140.39
	Power decrease (%)	37.11%	55.75%	56.52%	51.69%

19. Supplementary Table 7

Hardware Utilization on VCU128 FPGA

Component	LUT	FF	BRAM	URAM	DSP
**FP engine**	208K (16%)	176K (6.7%)	48 (2.4%)	192 (20%)	1284 (14.2%)
**BP engine**	268K (20.5%)	248K (9.5%)	36 (1.8%)	224 (23.3%)	2400 (26.6%)
**WG engine**	184K (14.1%)	132K (5.1%)	0	0	1048 (11.6%)
**Controller**	8K (0.6%)	8K (0.3%)	512 (25.4%)	0	0
**Interconnect**	289K (22.2%)	395K (15.2%)	519 (25.7%)	0	0
**Total used**	957K (73.4%)	959K (36.8%)	1115 (55.3%)	416 (43.3%)	4732 (52.4%)

20. Supplementary Table 8

(a) Comparison of PyTorch, JAX, and our custom IRs for SNNs

Model	Pytorch	JAX	Ours
Spiking ResNet-18	28.5MB	48K	4KB
Spiking ResNet-50	141MB	65K	11KB
Spiking VGG-16	209MB	37K	3KB

(b) Comparison of PyTorch/JAX IRs between ANNs and SNNs

Model	ANN (PyTorch)	SNN (PyTorch)	ANN (JAX)	SNN (JAX)
ResNet-18	11KB	28.5MB	16K	48K
ResNet-50	26KB	141MB	48K	65K
VGG-16	8KB	209MB	14K	37K

21. Supplementary Table 9

PVT Variation of Multi-Core Neuromorphic Architecture

Corners	Power of 32-Core Architecture (W)
tt_0.9V_25°C	14.493
ss_0.81V_125°C	25.254
ss_0.81V_-40°C	9.503
ff_1.05V_-40°C	22.620

22. Supplementary Table 10

Performance Comparison of the Architecture on FPGAs and Chip Taped Out

	FPGA	Chip
Cores	20	32
NoC Topology	2x10 (2D mesh)	4x8 (2D mesh)
Engine Clock(MHz)	150	500
Router Clock (MHz)	200	667
Inter-core Bandwidth(Gbps)	50 (within FPGA),<br>100 (inter- FPGAs, 2 core – 2 core)	170
Routing Strategy	X-Y Routing	X-Y Routing

23. Supplementary Table 11

Time to Achieve Convergence in Our Architecture

**Training Mode**	Fine-Tuning	Fine-Tuning	Full Training
**Dataset**	Traffic Sign Classification and Recognition	CIFAR-10	CIFAR-10
**Network**	3-layer Spiking CNN	Spiking ResNet-18	Spiking ResNet-18
**Time until Convergence**	9.29s	9.19min	2.30h

24. Supplementary Table 12

Representative Convolutional Spiking Neural Networks

	**Method**	**Year**	**Publication**	**Model Structure**	**FP16/BF16**
1	PLIF	2021	CVPR [18]	Simple CNNs	n/a
2	SEW	2021	NeurIPS [19]	ResNet-18, -34, -50, -101, -152	n/a
3	BNTT	2021	Front. Neurosci. [20]	VGG-9, -11	n/a
4	STBP-tdBN	2021	AAAI [21]	ResNet-19, -34, -50	n/a
5	DSpike	2021	NeurIPS [22]	ResNet-18, -34; VGG-16	n/a
6	Diet-SNN	2021	TNNLS [23]	VGG-16; ResNet-20	n/a
7	Tandeom	2021	TPAMI [24]	VGG-11, -16	n/a
8	Spiking ResNet	2021	TNNLS [25]	ResNet-18, -34, -50	n/a
9	TTRBR	2022	Neural Networks [26]	ResNet-18, -20, -32, -56, -110	n/a
10	LTL	2022	NeurIPS [27]	VGG-11, -16; ResNet-20	n/a
11	RecDis-SNN	2022	CVPR [28]	ResNet-19, -34; VGG-16	n/a
12	NDA	2022	ECCV [29]	VGG-11, -19; ResNet-19, -34	FP16
13	Temporal Pruning	2022	ECCV [30]	VGG16	n/a
14	TET	2022	ICLR [31]	ResNet-19, -34; VGGSNN	n/a
15	n/a	2022	AAAI [32]	VGG-16; ResNet-18, -20	n/a
16	QCFS	2022	ICLR [33]	VGG16; ResNet-18, -20, -34	n/a
17	DSR	2022	CVPR [34]	ResNet-18, -34; VGG-11	FP16
18	OTTT	2022	NeurIPS [35]	VGG-7; ResNet-34	FP16
19	LATS	2023	ICLR [36]	ResNet-18, -50	FP16
20	SML	2023	ICML [37]	ResNet-18, -19, -34; VGGSNN	FP16
21	PSN	2023	NeurIPS [38]	ResNet-18, -34; VGGSNN	FP16
22	SSF	2023	ICCV [39]	ResNet-18, -34; VGG-11	n/a
23	Dual-Phase	2023	TNNLS [40]	ResNet-18, -20, -34; VGG-16	n/a
24	Fast-SNN	2023	TPAMI [41]	VGG-11, 16; ResNet-18	n/a
25	Attention SNN	2023	TPAMI [42]	ResNet-18, -34, -104	FP16
26	MPBN	2023	ICCV [43]	ResNet-19, -20, -34; VGG-16	n/a
27	KDSNN	2023	CVPR [44]	ResNet-18; VGG-11	n/a
28	SLTT	2023	ICCV [45]	ResNet-18, -34, -50, -101; VGG-11	FP16
29	LocalZO	2023	NeurIPS [46]	ResNet-19, -34; VGGSNN	n/a

30	IM-LIF	2024	IEEE TETCI [47]	ResNet-19; VGGSNN	n/a
31	Parameter Calibration	2024	IJCV [48]	VGG-16; ResNet-20, -34	n/a
32	LM-H	2024	ICLR [49]	ResNet-18, -34, -50; VGG-16	FP16
33	BKDSNN	2024	ECCV [50]	ResNet-19, -34, -50	FP16
34	T-RevSNN	2024	ICML [51]	ResNet-18	FP16
35	n/a	2024	CVPR [52]	ResNet-34, -50, -19, -20	n/a
36	FSTA-SNN	2025	AAAI [53]	ResNet-18, -34	FP16

References

[1] Pehle, C. et al. Norse - A deep learning library for spiking neural networks. https://doi.org/10.5281/zenodo.4422025 (2021).

[2] Fang, W. et al. SpikingJelly: An open-source machine learning infrastructure platform for spike-based intelligence. *Sci. Adv.* **9**, eadi1480 (2023).

[3] Eshraghian, J. K. et al. Training spiking neural networks using lessons from deep learning. *Proc. IEEE* **111**, 1016–1054 (2023).

[4] Sheik, S. et al. SINABS: A simple Pytorch based SNN library specialised for Speck. https://github.com/synsense/sinabs (2023).

[5] Heckel, K. M. et al. Spyx: A library for just-in-time compiled optimization of spiking neural networks. Preprint at https://doi.org/10.48550/arXiv.2402.18994 (2024).

[6] Pedersen, J. E. et al. Neuromorphic intermediate representation: A unified instruction set for interoperable brain-inspired computing. *Nat. Commun.* **15**, 8122 (2024).

[7] Davison, A. P. et al. PyNN: a common interface for neuronal network simulators. *Front. Neuroinform.* **2**, 11 (2008).

[8] Gewaltig, M.-O. et al. NEST (Neural Simulation Tool). *Scholarpedia* **2**, 1430 (2007).

[9] Bekolay, T. et al. Nengo: a Python tool for building large-scale functional brain models. *Front. Neuroinform.* **7**, 48 (2014).

[10] Carnevale, N. T. et al. *The NEURON Book* (Cambridge University Press, 2006).

[11] Williams, M. G. K. et al. Lava - a software framework for neuromorphic computing. https://github.com/lava-nc/lava (2023).

[12] Amir, A. et al. Cognitive computing programming paradigm: A corelet language for composing networks of neurosynaptic cores. *Proc. Int. Jt. Conf. Neural Netw. (IJCNN),* 1–10 (2013).

[13] Muir, D. R. et al. Rockpool documentation. https://doi.org/10.5281/zenodo.3773845 (2019).

[14] Davies, M. et al. Loihi: A neuromorphic manycore processor with on-chip learning. *IEEE Micro* **38**, 82–99 (2018).

[15] Davies, M. et al. Advancing neuromorphic computing with Loihi: a survey of results and outlook. *Proc. IEEE* **109**, 911–934 (2021).

[16] Merolla, P. et al. A million spiking-neuron integrated circuit with a scalable communication network and interface. *Science* **345**, 668–673 (2014).

[17] Bos, H. et al. Sub-mW Neuromorphic SNN Audio Processing Applications with Rockpool and Xylo. *In: Embedded Artificial Intelligence: Devices, Embedded Systems, and Industrial Applications*, pp. 69–78, (2022).

[18] Fang, W. et al. Incorporating learnable membrane time constant to enhance learning of spiking neural networks. *Proc. Int. Conf. Comput. Vis. (ICCV)*, 1–10 (2021).

[19] Fang, W. et al. Deep residual learning in spiking neural networks. *Proc. Adv. Neural Inf. Process. Syst. (NeurIPS)* **34**, 21056–21069 (2021).

[20] Kim, Y. et al. Revisiting batch normalization for training low-latency deep spiking neural networks from scratch. *Front. Neurosci.* **14**, 592853 (2020).

[21] Zheng, H. et al. Going deeper with directly-trained larger spiking neural networks. *Proc. AAAI Conf. Artif. Intell. (AAAI)* **35**, 11062–11070 (2021).

[22] Li, Y. et al. Differentiable spike: Rethinking gradient-descent for training spiking neural networks. *Proc. Adv. Neural Inf. Process. Syst. (NeurIPS)* **34**, 1–12 (2021).

[23] Rathi, N. et al. Diet-snn: A low-latency spiking neural network with direct input encoding and leakage and threshold optimization. *IEEE Trans. Neural Netw. Learn. Syst.* **32**, 11 (2021).

[24] Wu, J. et al. Progressive tandem learning for pattern recognition with deep spiking neural networks. *IEEE Trans. Pattern Anal. Mach. Intell.* **44**, 7824–7840 (2021).

[25] Hu, Y. et al. Spiking deep residual network. *IEEE Trans. Neural Netw. Learn. Syst.* **34**, 1–15 (2021).

[26] Meng, Q. et al. Training much deeper spiking neural networks with a small number of time-steps. *Neural Netw.* **153**, 254–268 (2022).

[27] Yang, Q. et al. Training spiking neural networks with local tandem learning. *Proc. Adv. Neural Inf. Process. Syst. (NeurIPS)* **35**, 1–12 (2022).

[28] Guo, Y. et al. Recdis-snn: Rectifying membrane potential distribution for directly training spiking neural networks. *Proc. IEEE/CVF Conf. Comput. Vis. Pattern Recognit. (CVPR)*, 1–10 (2022).

[29] Li, Y. et al. Neuromorphic data augmentation for training spiking neural networks. *Proc. Eur. Conf. Comput. Vis. (ECCV)*, 1–17 (2022).

[30] Chowdhury, S. S. et al. Towards ultra low latency spiking neural networks for vision and sequential tasks using temporal pruning. *Proc. Eur. Conf. Comput. Vis. (ECCV)*, 1–17 (2022).

[31] Deng, S. et al. Temporal efficient training of spiking neural network via gradient re-weighting. *Proc. Int. Conf. Learn. Represent. (ICLR)* (2022).

[32] Bu, T. et al. Optimized potential initialization for low-latency spiking neural networks. *Proc. AAAI Conf. Artif. Intell. (AAAI)* **36**, 1–9 (2022).

[33] Bu, T. et al. Optimal ANN-SNN conversion for high-accuracy and ultra-low-latency spiking neural networks. *Proc. Int. Conf. Learn. Represent. (ICLR)* (2022).

[34] Meng, Q. et al. Training high-performance low-latency spiking neural networks by differentiation on spike representation. *Proc. IEEE/CVF Conf. Comput. Vis. Pattern Recognit. (CVPR)*, 12444–12453 (2022).

[35] Xiao, M. et al. Online training through time for spiking neural networks. *Proc. Adv. Neural Inf. Process. Syst. (NeurIPS)* **35**, 1–12 (2022).

[36] Chen, Y. et al. A unified framework for soft threshold pruning. *Proc. Int. Conf. Learn. Represent. (ICLR)* (2023).

[37] Deng, S. et al. Surrogate module learning: Reduce the gradient error accumulation in training spiking neural networks. *Proc. Int. Conf. Mach. Learn. (ICML)*, 1–21 (2023).

[38] Fang, W. et al. Parallel spiking neurons with high efficiency and ability to learn long-term dependencies. *Proc. Adv. Neural Inf. Process. Syst. (NeurIPS)* **36**, 1–15 (2023).

[39] Wang, J. et al. SSF: Accelerating training of spiking neural networks with stabilized spiking flow. *Proc. Int. Conf. Comput. Vis. (ICCV)*, 1–11 (2023).

[40] Wang, Z. et al. Toward high-accuracy and low-latency spiking neural networks with two-stage optimization. *IEEE Trans. Neural Netw. Learn. Syst.* **35**, 1–15 (2023).

[41] Hu, Y. et al. Fast-SNN: Fast spiking neural network by converting quantized ann. *IEEE Trans. Pattern Anal. Mach. Intell.* **45**, 14546–14562 (2023).

[42] Yao, M. et al. Attention spiking neural networks. *IEEE Trans. Pattern Anal. Mach. Intell.* **45**, 9393–9410 (2023).

[43] Guo, Y. et al. Membrane potential batch normalization for spiking neural networks. *Proc. Int. Conf. Comput. Vis. (ICCV)*, 1–10 (2023).

[44] Xu, Q. et al. Constructing deep spiking neural networks from artificial neural networks with knowledge distillation. *Proc. IEEE/CVF Conf. Comput. Vis. Pattern Recognit. (CVPR)*, 1–10 (2023).

[45] Meng, Q. et al. Towards memory- and time-efficient backpropagation for training spiking neural networks. *Proc. Int. Conf. Comput. Vis. (ICCV)*, 1–11 (2023).

[46] Mukhoty, B. et al. Direct training of snn using local zeroth order method. *Proc. Adv. Neural Inf. Process. Syst. (NeurIPS)* **36**, 1–14 (2023).

[47] Lian, S. et al. Im-lif: Improved neuronal dynamics with attention mechanism for direct training deep spiking neural network. *IEEE Trans. Emerg. Top. Comput. Intell.* **8**, 2075–2085 (2024).

[48] Li, Y. et al. Error-aware conversion from ann to snn via post-training parameter calibration. *Int. J. Comput. Vis.* **132**, 3586–3609 (2024).

[49] Hao, Z. et al. A progressive training framework for spiking neural networks with learnable multi-hierarchical model. *Proc. Int. Conf. Learn. Represent. (ICLR)* (2024).

[50] Xu, Z. et al. Bkdsnn: Enhancing the performance of learning-based spiking neural networks training with blurred knowledge distillation. *Proc. Eur. Conf. Comput. Vis. (ECCV)*, 106–123 (2024).

[51] Hu, J. et al. High-performance temporal reversible spiking neural networks with O(l) training memory and O(l) inference cost. *Proc. Int. Conf. Mach. Learn. (ICML)*, 1–21 (2024).

[52] Shen, G. et al. Are conventional snns really efficient? a perspective from network quantization. *Proc. IEEE/CVF Conf. Comput. Vis. Pattern Recognit. (CVPR)*, 27538–27547 (2024).

[53] Yu, K. et al. Fsta-snn: Frequency-based spatial-temporal attention module for spiking neural networks. *Proc. AAAI Conf. Artif. Intell. (AAAI)* **39**, 22227–22235 (2025).
